# Research hotspots and trends in the treatment of postoperative pain with ropivacaine: A bibliometric analysis from 2003 to 2022

**DOI:** 10.1097/MD.0000000000040707

**Published:** 2024-12-06

**Authors:** Bin Yin, Hexiang Wang, Xiaoming Zhao, Liwen Zhang, Yuanyuan Yu

**Affiliations:** a Department of Pulmonary Disease, Qingdao Hiser Hospital Affiliated of Qingdao University (Qingdao Traditional Chinese Medicine Hospital), Qingdao, China; b Department of Pathology, Qingdao Hiser Hospital Affiliated of Qingdao University (Qingdao Traditional Chinese Medicine Hospital), Qingdao, China; c Department of Anesthesiology, Qingdao Hiser Hospital Affiliated of Qingdao University (Qingdao Traditional Chinese Medicine Hospital), Qingdao, China; d Major in Anesthesiology, Medical Department, Qingdao University, Qingdao, China.

**Keywords:** ropivacaine, chronic postoperative pain, bibliometric, literature, treatment

## Abstract

**Background:**

The aim of this study is to explore the current study status and progression of the effects of ropivacaine in local analgesia by using bibliometric methods.

**Methods:**

Published articles related to the treatment of postoperative pain with ropivacaine from 2003 to 2022 were retrieved from the Web of Science database. Detailed information such as authors, keywords, journals, countries, institutions and references were analyzed by bibliometric methods and visualized by VOSviewer.

**Results:**

A total of 1150 articles published in the treatment of postoperative pain with ropivacaine were included in the bibliometric analysis. From 2003 to 2022, the number of articles was on the rise and increased rapidly after 2018. The United States published the highest number of articles and Ilfeld ranked first in the number of papers (35) and citation frequency (1670). Journal of *Anesthesiology* had the highest average number of citations per article (89.43). There is a close cooperation among countries especially centering China and the United States at their core, universities are the main institutions in publishing articles. The most cited literature was published by Knudsen whose study major in finding out the effects of ropivacaine and bupivacaine to the central nervous system. Among the top 10 high frequency keywords, ropirvacaine and postoperative anesthesia appeared most frequently.

**Conclusion:**

The application of ropivacaine to postoperative anesthesia attracts growing interest from the scholars all over the world and was the future research hotspot in treating postoperative pain.

## 1. Introduction

Pain is an unpleasant sensory and emotional experience related to genuine or potential tissue damage, so how to control and manage the postoperative pain are the most concerned questions of both patients and surgeons. Due to surgical intervention, postoperative pain frequently happens to the patients and it could delay postoperative recovery even lead to an increased risk of wound infection, respiratory and cardiovascular complications, all of which decrease patients’ fulfillment. Intractable acute pain is called chronic postoperative pain (CPSP) which is defined as a kind of postoperative pain persevered for at least 3 months. Different from preoperative pain, CPSP is confined to the surgical site with no other apparent etiology. Thus, CPSP can significantly affect the patient’s quality of life and daily activities including sleep and emotional interference, CPSP can not only reduce the patient’s quality of life but also increase the patient’s financial burden.^[1]^ Therefore, effective prevention of chronic postoperative pain is an important purpose of modern clinical treatment.

Opioid analgesics were once considered the traditional approaches to prevent postoperative pain by targeting the central mechanisms involved in pain perception. Compared with the single-drug approach, the application of multi-mode analgesia is widely acknowledged as the mainstream treatment of pain. Multi-mode analgesia refers to the synchronous utilization of several different analgesics or strategies to reduce the activity of pain receptors and decrease the local hormonal response to injury. It has been demonstrated that multi-mode analgesia not only effectively alleviates the pain but also reduces dependence on specific drugs or mechanisms.^[2]^ Besides, regional anesthesia is gaining increasing attention in multi-mode analgesia, the study on the prevention of postoperative chronic pain by regional anesthesia has also become a hotspot in recent years.

Ropivacaine, a kind of long-acting amide local anesthetics, is a pure S-enantiomer with high pKa and low lipid solubility. Compared with bupivacaine, ropivacaine has lower toxicity and higher safety in the central nervous system and cardiovascular system and can produce fewer motor blocks.^[3,4]^ In recent years, with the development of nerve block techniques, ropivacaine has become increasingly widely used in postoperative analgesia. Furthermore, many studies have demonstrated the effectiveness of ropivacaine in treating postoperative pain. However, there are few studies on the general characteristics and development trend of ropivacaine in the treatment of postoperative pain, which is not conducive for researchers to comprehensive understand the status quo and development trend of this field, moreover, it is difficult for researcher to grasp the future direction.

Bibliometric, which first appeared in the early 20th century, is a subject that uses mathematical and statistical techniques to quantify and analyze literature. It is a comprehensive knowledge system by which we could obtain detailed information such as authors, keywords, journals, countries, institutions, references and so on, besides, we could obtain graphical and visual results with the assistance of modern computer techniques to supplement literature analysis. Thus, bibliometric help us understand the research status and development trend from different perspective. The purpose of this study is to apply the bibliometric method to analyzing the literature related to the treatment of postoperative pain with ropivacaine, in the meantime, systematically understand the research progress and future development directions in this field.

## 2. Methods

### 2.1. Literature sources and retrieval methods

The study was conducted in accordance with the principles of the Declaration of Helsinki, and the study protocol was approved by the Ethics Committee of Qingdao Traditional Chinese Medicine Hospital (Qingdao Hiser Hospital). Because of the retrospective nature of the study, patient consent for inclusion was waived.

In this study, Web of Science (core set) was selected as the data source, SCI-EXPANDED and SSCI were selected as the indexes to ensure the comprehensive and accurate retrieval data. An advanced search was performed with the search formula TS = “Postoperative analgesia” AND TS = Ropivacaine. The time span was from January 1, 2003 to December 31, 2022. The language type was limited to English. The literature types were limited to “article or review.” All the titles, authors, abstracts and citations of the literature were derived in TXT format by using the above formula, excluding repetitive literature.

### 2.2. Research methods

**Figure 1.**
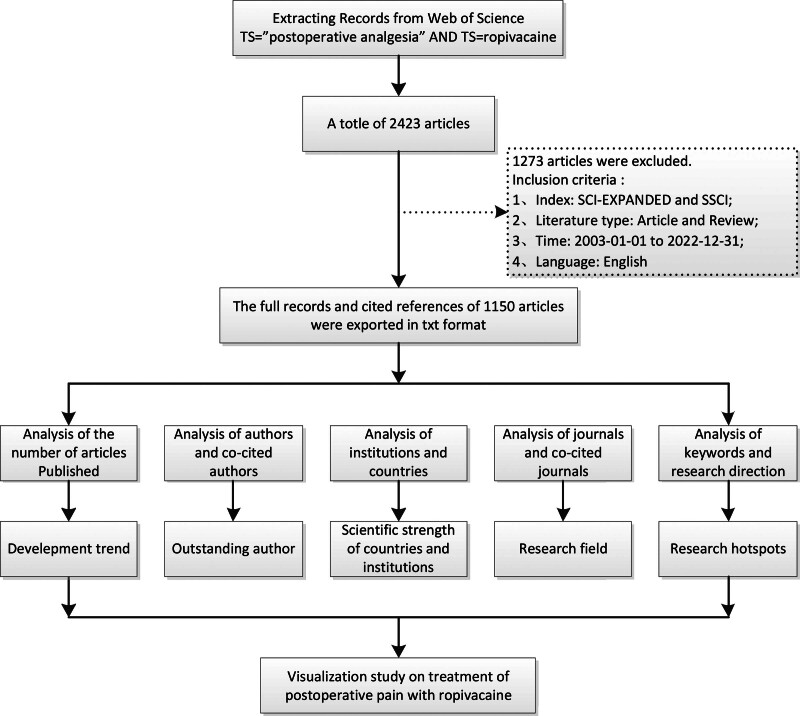
Analysis of the treatment of postoperative pain with ropivacaine.

VOSviewer is an analysis software used to construct and view bibliometric maps especially emphasize on the graphical representation of bibliometric maps and provide 2 import modes such as text set and network form, as well as 4 kinds of map browsing modes such as tag view, density view, cluster view and scatter view.^[5]^ VOSviewer has strong visualization ability and is suitable for large-scale sample data. In this study, we used VOSviewer to visualized analyze the authors, countries, institutions, journals, keywords, co-cited journals and literatures. Figure 1 illustrates the detailed analysis process of this study.

## 3. Results

We retrieved 1150 articles originated from 5165 authors who were from 1267 institutions in 59 countries. The articles which cited 17,488 references from 2877 journals were published in 284 journals.

### 3.1. Analysis of the number of articles published

**Figure 2.**
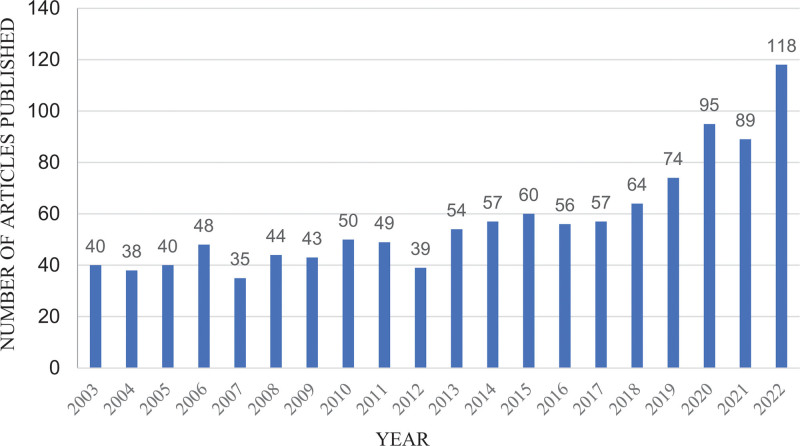
The number of annual articles published related to the treatment of postoperative pain with ropivacaine.

The time distribution of the articles published in the treatment of postoperative pain with ropivacaine was shown in Figure 2. Obviously, the number was on the rise, particularly after 2018, the number increased rapidly and stabilized at more than 80 from 2020 to 2022, and reached 118 in 2022,which showed that it is a research hotspot of the treatment of postoperative pain with ropivacaine and the study has attracted more and more attentions from scholars.

### 3.2. Analysis of authors and co-cited authors

By analyzing the authors and co-cited authors, we learned the leading scholars and core research strength in this field. Price pointed out that half of the articles were published by a group of high-productivity authors, and the number of high-productivity authors was approximately equal to the square root of the total number of authors, that is


$$\begin{array}{l} \underset{m+1}{\overset{I}{\sum }}\;n\left(x\right)=\sqrt{N} \end{array}$$

*m* is the minimum number of articles published by core authors; *I* = n_max_, is the number of articles published by the highest yield author in the field; n (*x*) is the number of authors who wrote × articles; N is the total number of authors. By using the VOSviewer statistics, n_max_ =3 5, meanwhile, according to Price’s law, the minimum number of articles published by core authors in a field is *m* = 0.749 × $\sqrt{n_{max}}$, thus 4.4 was concluded from the formula calculation. We defined the author who had >5 articles (including 5 articles) as the core author in the field, thus there were 81 core authors with totally 602 articles, accounting for 52.35% of the total number of articles published. This result achieved half (50%) of the criteria proposed by Price and basically in line with the Price’s law by substituting value. Therefore, a relatively stable author cooperation group has formed in the field of treating postoperative pain with ropivacaine. Table 1 listed the top 5 authors in the number of publications in treating postoperative pain with ropivacaine.

**Table 1:** Top 5 authors in the number of articles published in treating postoperative pain with ropivacaine.

Rank	Author	Documents	Citations	Average article citations	Country
1	Ilfeld, BM	35	1607	45.91	USA
2	Capdevila, X	18	1032	57.34	France
3	Mariano, ER	18	763	42.39	USA
4	Loland, VJ	15	692	46.13	USA
5	Enneking, FK	13	469	36.08	USA

Among the top 5 authors published articles in treating postoperative pain with ropivacaine, 4 authors are from the United States, demonstrating the strong scientific research strength of the United States in this research field. Ilfeld, an American scholar, ranked first and published 35 articles from 2003 to 2022, his published articles received 1607 citations and the average number of citations per article is 45.91. Ilfeld works at the University of California San Diego, mainly studies on the effect of treating postoperative pain with ropivacaine through various modes of peripheral nerve block including intercalary brachial plexus block,^[6]^ paravertebral block and femoral block.^[7,8]^ Capdevila, a French scholar, ranked second and published 18 articles from 2003 to 2022, his published articles received 1032 citations, and the average number of citations per article is 57.34. Capdevila works at CHU de Montpellier, mainly studies on the effect of peripheral nerve block in children’s postoperative analgesia.^[9]^

**Figure 3.**
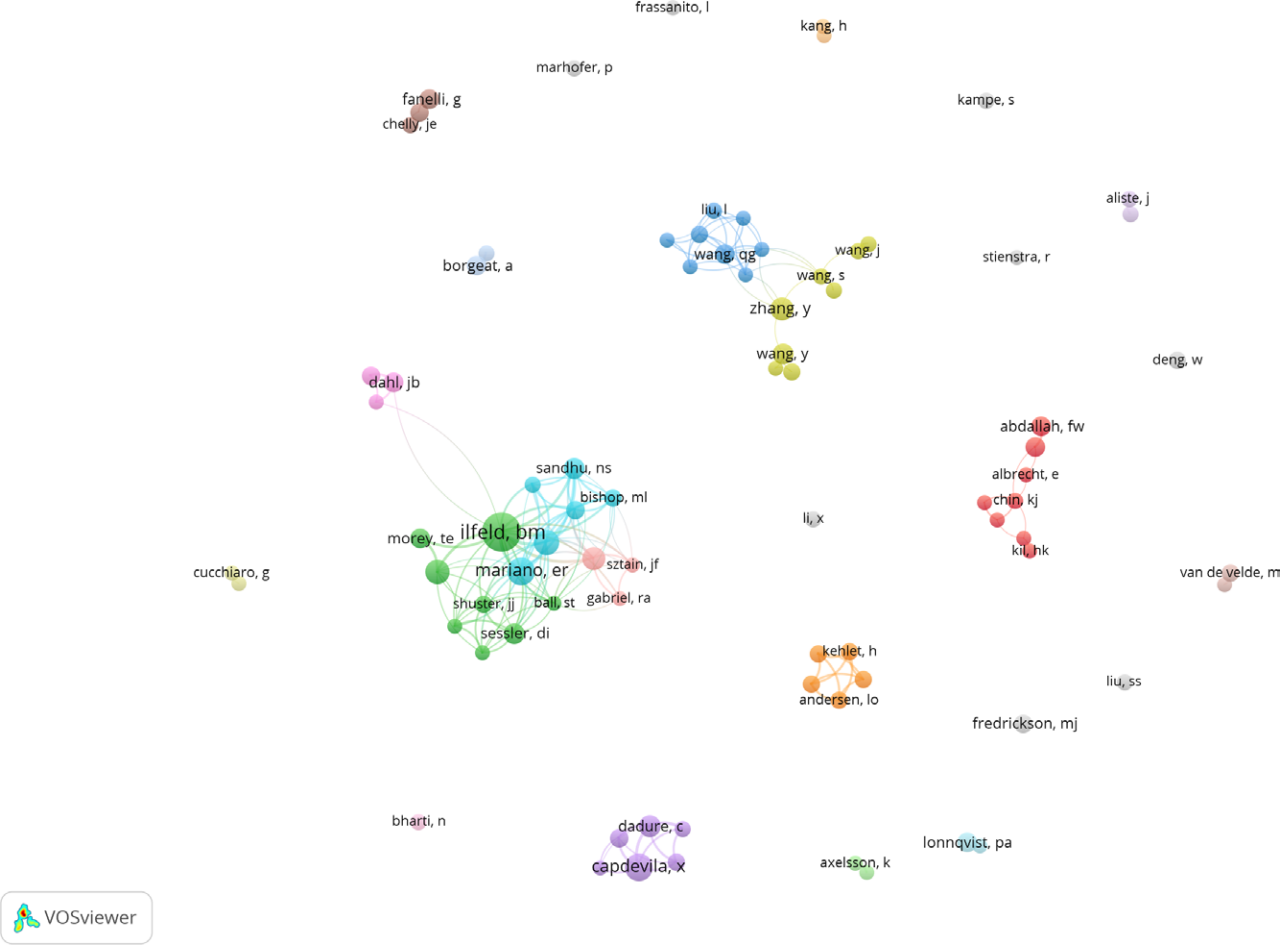
Cooperative cluster view of core authors.

A collaborative clustering view of 81 core authors was shown in Figure 3. It can be seen that many stable cooperative groups have been formed and group around Ilfeld is the largest one, especially 4 out of the top 5 authors contributed articles on treating postoperative pain with ropivacaine are members of this group which illustrates the extremely close collaboration among high-productivity authors in this field (Fig. 3).

**Figure 4.**
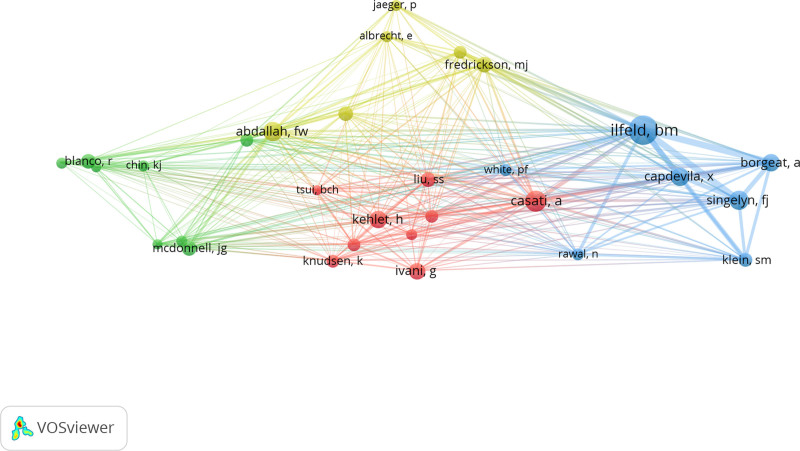
Network clustering view of the co-cited authors.

Subsequently, we brought 12,529 co-cited authors into the analysis and set the lowest co-cited times to 50. There into, top 30 authors were retained for a visualized analysis and the result was shown in Figure 4. Moreover, top 5 authors in the co-cited times were presented in Table 2.

**Table 2:** Top 5 authors in the co-cited times in the treatment of postoperative pain with ropivacaine.

Rank	Author	Co-cited	Country
1	Ilfeld, BM	386	USA
2	Casati, A	254	Italy
3	Singelyn, FJ	164	France
4	Abdallah, FW	162	Canada
5	Capdevila, X	155	France

Among the co-cited authors, it is still the American scholar Ilfeld whose published articles achieved the most cited times. His most cited article described the relationship between continuous peripheral nerve block in the lower limbs and patients’ fall down who had taken a knee or hip arthroplasty.^[10]^ At the same time, we can see from Figure 4 that there is a close relationship among the top 5 authors which indicates a close cooperation among influential authors in this field.

### 3.3. Analysis of journals

According to the statistical analysis, in the past 2 decades, the journals in which most of the articles related to our study published belong to the discipline of anesthesia. Table 3 presented the top 10 journals in the number of articles published, it is evidently, journal of *Anesthesia and Analgesia* (103) ranked first, journal of *Regional Anesthesia and Pain Medicine* (80) and *British Journal of Anesthesia* (45) ranked second and third respectively. Morever, journal of *Anesthesiology*,a leading journal in the discipline of anesthesia, had the highest average number of citations per article(89.43). This journal had 18 related articles published and was cited 2504 times.

**Table 3:** Top 10 journals in the number of publications in the treatment of postoperative pain with ropivacaine.

Rank	Source	Documents	Citations	Average citations/publication
1	Anesthesia and Analgesia	103	4867	47.25
2	Regional Anesthesia and Pain Medicine	80	2964	37.05
3	British Journal of Anaesthesia	45	3054	67.87
4	Pediatric Anesthesia	40	805	20.125
5	BMC Anesthesiology	38	290	7.63
6	Journal of Clinical Anesthesia	35	809	23.11
7	Journal of Pain Research	31	202	6.52
8	European Journal of Anaesthesiology	29	733	25.28
9	Anesthesiology	28	2504	89.43
10	Medicine	27	276	10.22

### 3.4. Analysis of countries

**Figure 5.**
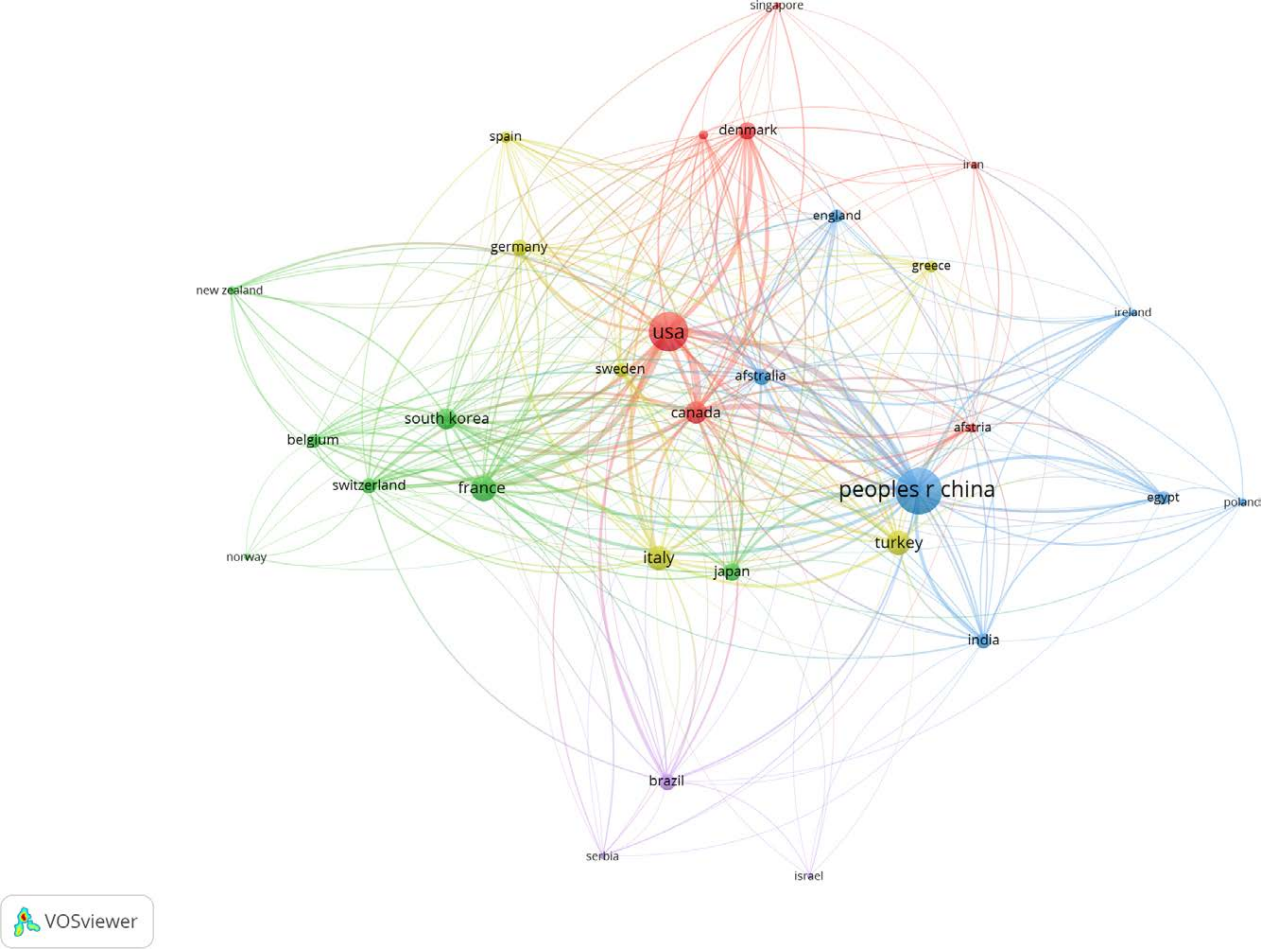
Network clustering view of countries.

In order to understand which country contributed most to the field of treating postoperative pain with ropivacaine, we used VOSviewer to visualize countries in the number of published articles >5. As shown in Figure 5, there is a close cooperation among countries especially center China and the United States at their core. However, the distribution of the countries with articles published is imbalance. Due to most of the articles are published by scholars from a few countries, the top effect phenomenon is quite obvious.

Furthermore, we conducted analysis on the high productive countries in this field. Table 4 presented the top 10 countries in the number of published articles in this field. We can see that Chinese scholars contributed the most articles (259), accounting for 22.5% of the total number of articles published in this field, however, they were cited only 2210 times and the average number of citations per article was only 8.53. Following by the United States, contributed 190 published articles to this field. They were cited 6212 times and the average number of citations per article was 32.69. It is noteworthy that Denmark contributed only 36 published articles which were cited 1996 times and the average number of citations per article was 55.44.

**Table 4:** Top 10 countries in the number of publications in treating postoperative pain with ropivacaine.

Rank	Country	Documents	Citations	Average citations/publication
1	China	259	2210	8.53
2	USA	190	6212	32.69
3	France	77	2623	34.06
4	Italy	70	1354	19.34
5	Turkey	70	864	12.34
6	Canada	62	3239	52.24
7	South-Korea	57	909	15.95
8	Japan	39	563	14.44
9	Denmark	36	1996	55.44
10	Germany	34	896	26.35

### 3.5. Analysis of institutions

We took statistical analysis on the institutions the authors belong to, Table 5 listed the top 10 institutions in the number of articles published. It is obviously that universities are the main institutions in publishing articles in this research field. Among them, university of *California San Diego* has the largest number of articles published, while university of *Toronto* has the highest citations and the average number of citations per article. In terms of the distribution of the countries where the institutions are located, Top 10 institutions are covered by 4 countries. To be exactly, there are 5 institutions in the United States, 3 institutions in the China, 1 institution in the Canada and Denmark respectively.

**Table 5:** Top 10 institutions in the number of publications in treating postoperative pain with ropivacaine.

Rank	Organization	Country	Documents	Citations	Average citations/publication
1	Univ Calif San Diego	USA	28	1109	39.61
2	Univ Toronto	Canada	24	1388	57.83
3	Univ Florida	USA	19	738	38.84
4	Univ Copenhagen	Denmark	18	609	33.83
5	Sichuan Univ	China	16	129	8.06
6	Cleveland Clin	USA	15	666	44.4
7	Wenzhou Med Univ	China	13	132	10.15
8	Capital Med Univ	China	13	63	4.85
9	Univ Pittsburgh	USA	13	414	31.85
10	Ohio State Univ	USA	12	133	11.08

### 3.6. Analysis of co-cited journals and literatures

**Figure 6.**
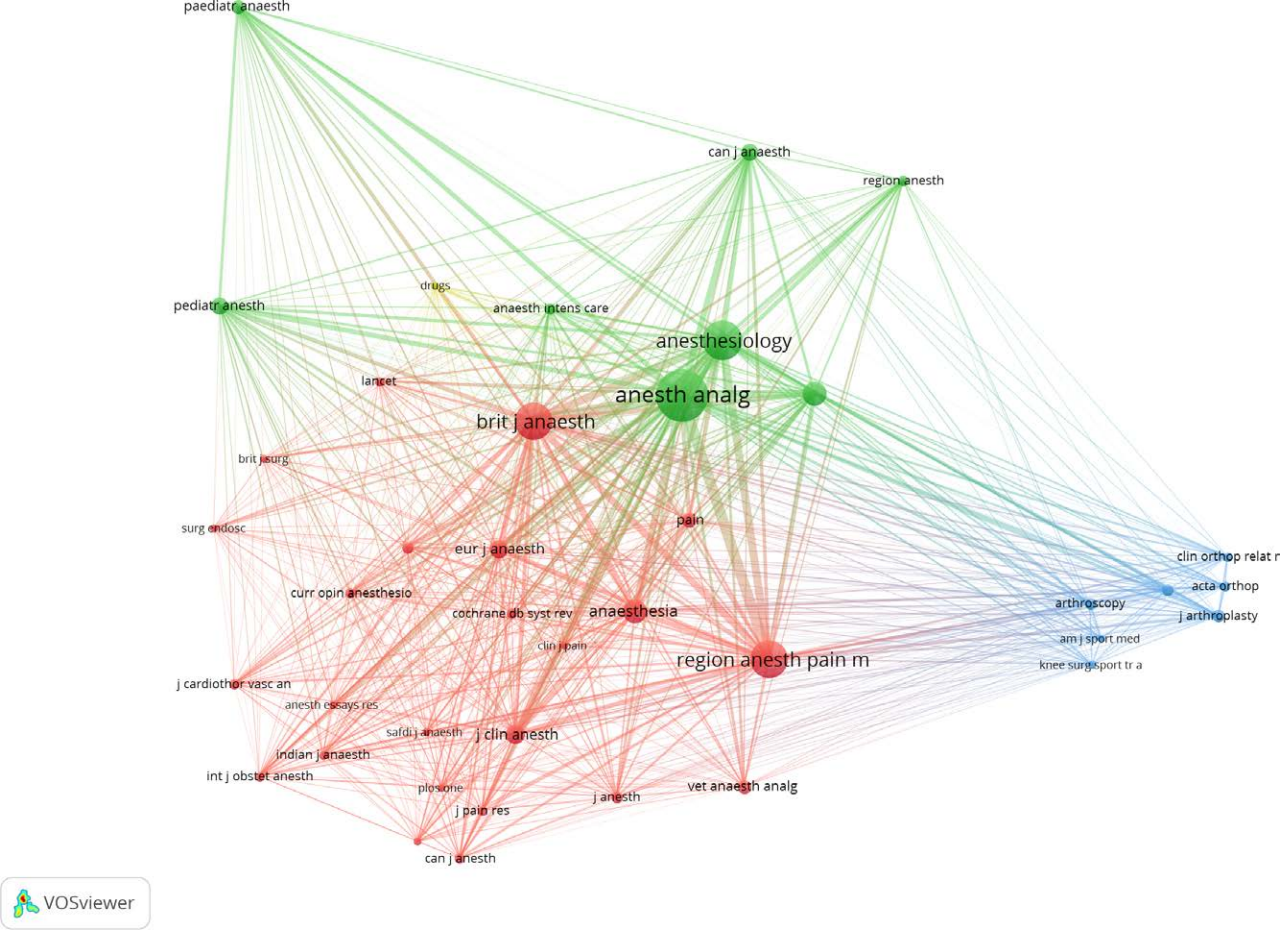
Network clustering view of the co-cited journals.

In order to understand the high frequency cited articles and the journals published them in this research field, we drew the co-cited map of co-cited journals by VOSviewer. First of all, we set the minimum co-citation times to 110, only 40 journals were retained for the co-citation analysis of the cited journals. As shown in Figure 6.

The co-cited network of journals is divided into 4 clusters and represented by 4 colors. The top 3 journals with high citations were *Anesthesia & Analgesia* (4558), *Anesthesiology* (2600) and *British Journal of Anaesthesia* (2358) respectively. All 3 journals are the leading journals in the discipline of anesthesia.

Among the 4 clusters, journals of red and green clusters are mainly in the clinical journals and mainly in the field of anesthesia and pain whose content covering general and regional anesthesia, intensive care and pain treatment, etc. Journals of blue cluster are mainly in the field of orthopedics, including basic and clinical research in orthopedics and related subspecialty, while journals of yellow cluster are pharmaceutical journals which provide reviews and research articles on the most important aspects of clinical pharmacology and therapeutics to promote optimal medical treatment.

Subsequently, we analyzed the co-cited literatures by VOSviewer. Table 6 listed the top 5 literatures in the number of citations from 2003 to 2022. As shown in the table, top 3 literatures were all published before 2000 as well as the most cited literature was published by Knudsen et al^[11]^ whose study major in finding out the effects of ropivacaine and bupivacaine to the central nervous system and cardiovascular system. Nevertheless the literatures ranked second and third respectively were the study all related to postoperative analgesia of knee joint.^[12,13]^

**Table 6:** Top 5 co-cited literatures related to the treatment of postoperative pain with ropivacaine.

Rank	Title	Author	Year	Citations
1	Central nervous and cardiovascular effects of i.v. infusions of ropivacaine, bupivacaine and placebo in volunteers	Knudsen K	1997	72
2	Effects of perioperative analgesic technique on the surgical outcome and duration of rehabilitation after major knee surgery	Capdevila X	1999	57
3	Effects of intravenous patient-controlled analgesia with morphine, continuous epidural analgesia, and continuous 3-in-one block on postoperative pain and knee rehabilitation after unilateral total knee arthroplasty	Singelyn FJ	1998	51
4	The analgesic efficacy of transversus abdominis plane block after cesarean delivery: A randomized controlled trial	Mcdonnell JG	2008	42
5	Efficacy of continuous wound catheters delivering local anesthetic for postoperative analgesia: A quantitative and qualitative systematic review of randomized controlled trials	Liu SS	2006	40

**Figure 7.**
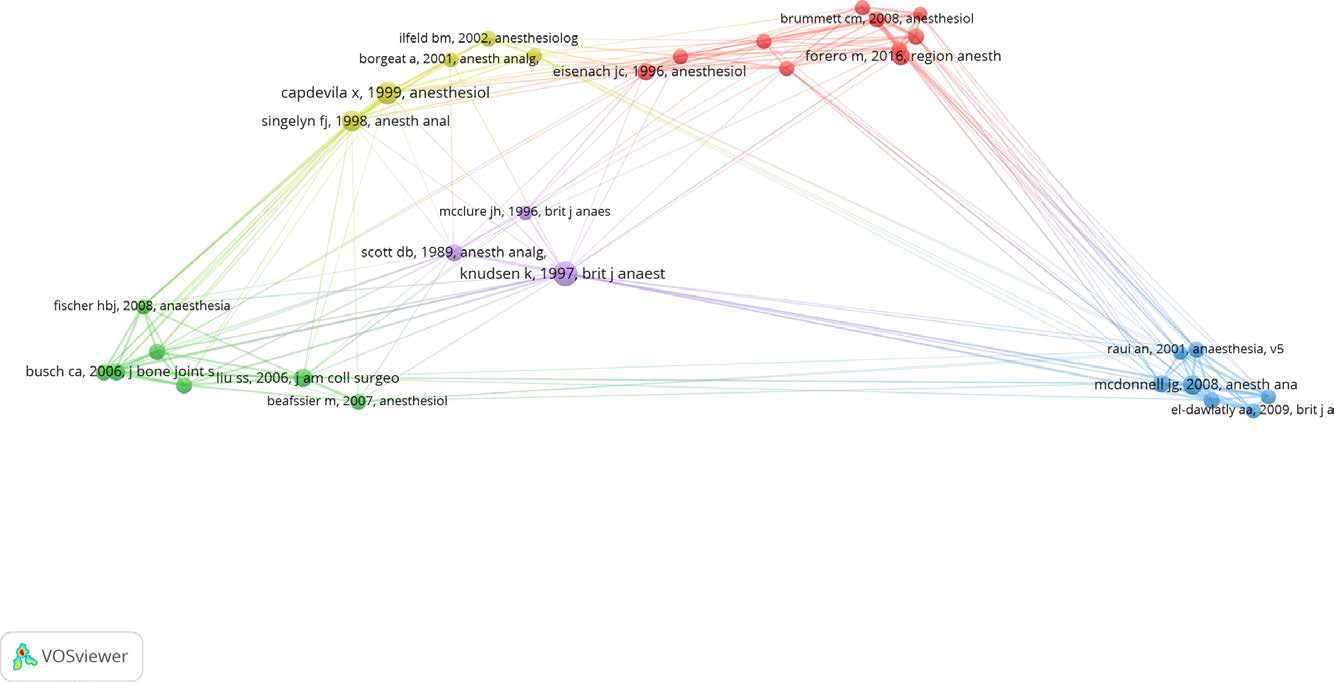
Network clustering view of high-co-cited literatures.

We then used VOSviewer to draw the cited map of references and set the minimum number of co-citations to 25, eventually retained 32 references for co-cited analysis. The map of relationship was shown in Figure 7.

We can see from Figure 7 that the cited literature co-cited network be divided into 5 clusters which corresponding to 5 colors in the map. The meaning of color was shown as follows: purple clustering mainly represents the literatures on comparing the intensity of toxicity between ropivacaine and bupivacaine; yellow clustering represents the study of relevant literatures on the joint nerve block; blue clustering represents the study of literatures on transverse ventral plane block; green clustering represents the study of literatures on multimodal analgesia; red clustering represents the study of literatures on analgesic effects after adding adjuvants to local anesthetics.

By analyzing the years the high co-cited literatures published, we found that most of the high co-cited literatures were published from 2006 to 2010, approximately a total of 15 literatures, accounting for 46.875% of the totality. The literatures within the purple cluster were published before 2000 which indicated that the comparison of effect between ropivacaine and bupivacaine was a hot research topic at that time. The yellow cluster was mainly literatures published around 2000, during which time the block of the joint nerve was concerned by scholars. While the blue and green clusters were mainly literatures published from 2005 to 2010, at that time, the application of a transverse peritoneal block and the study of multimodal analgesia had been the focus in this field. However, the red cluster was mainly literatures published since 2010, with the development of science and technology, the study on the different analgesic effect after adding different adjuvants to local anesthetics has become the focus of scholars in recent years.

### 3.7. Analysis of research direction

**Figure 8.**
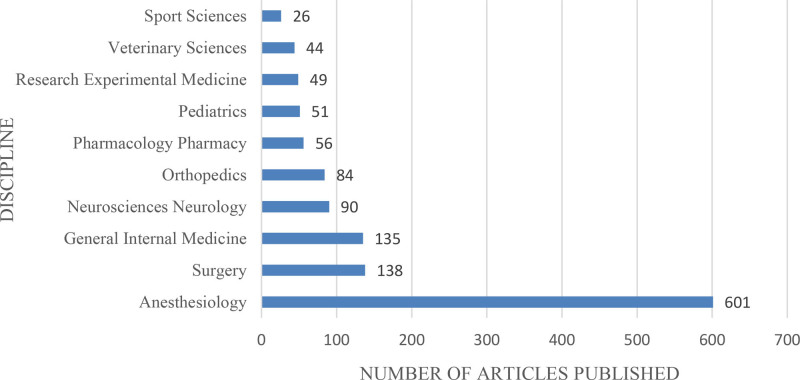
Top 10 research directions of literatures related to the treatment of postoperative pain with ropivacaine.

We took statistical analysis on the research directions in 1150 articles, a total of 55 research directions were found. As shown in Figure 8, among the top 10 research directions, there are 601 articles related to the discipline of anesthesiology and accounted for 52.261% of the total number of articles.

### 3.8. Analysis of keywords

**Figure 9.**
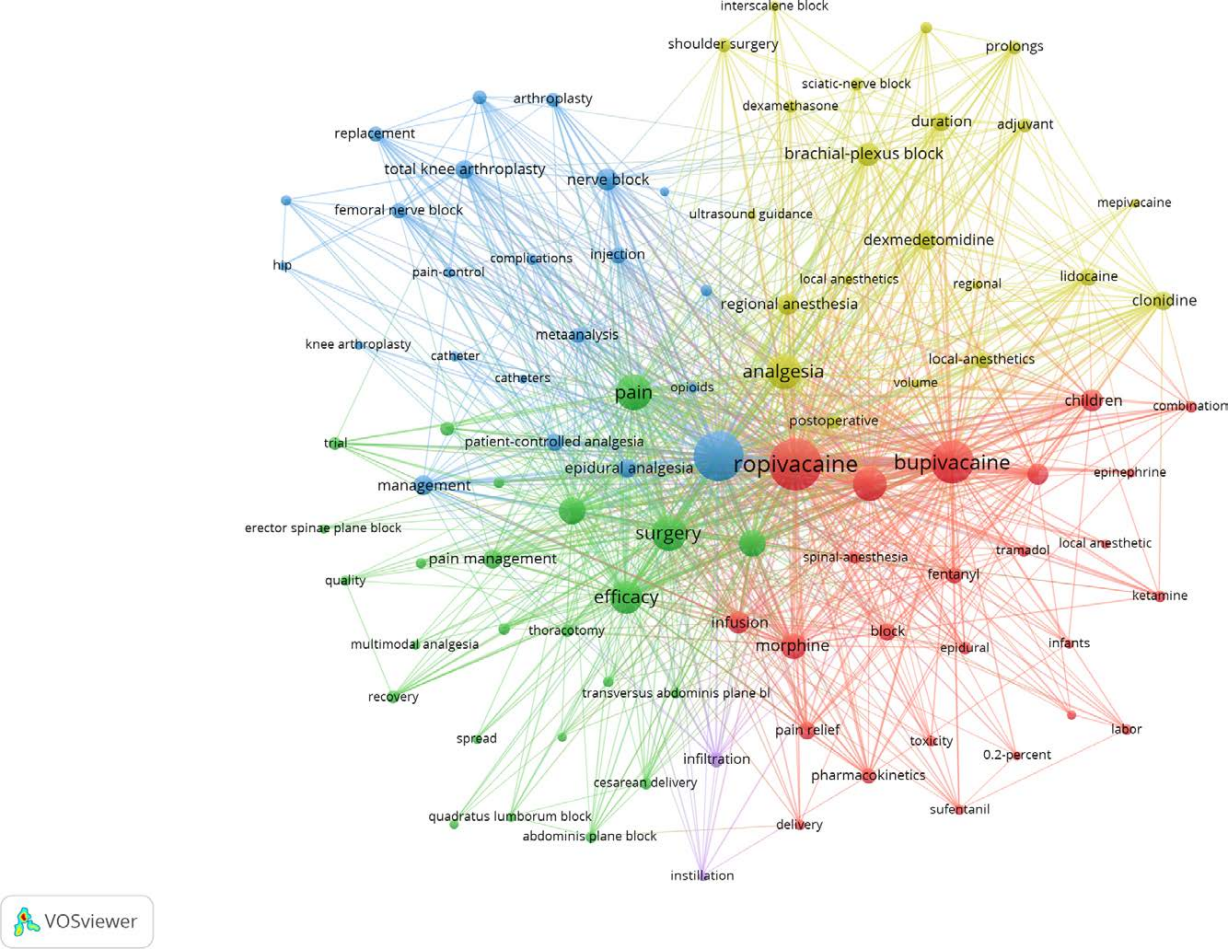
Network clustering view of keywords.

**Figure 10.**
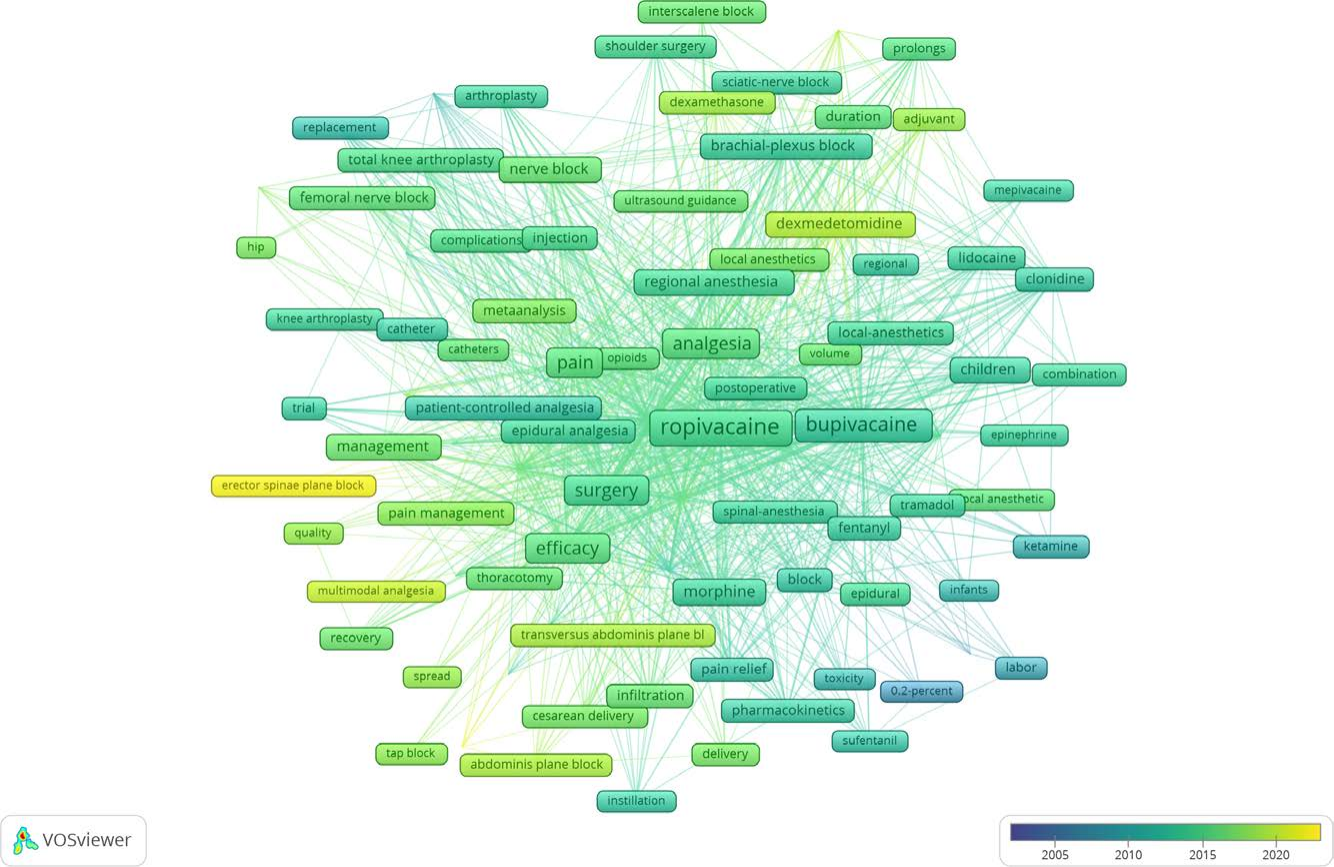
Network tag view of keywords.

Through the co-occurrence analysis of keywords, we can learn the research hotspot in a certain scientific field. We used VOSviewer to draw keywords network views from 1150 articles, according to Price’s Law, the core keywords with frequency ≥ 19 times were selected to visualize, the result was shown in Figure 9. We can see the larger the circular node, the more frequently the keywords appear, and more likely to represent the research hotspots in the field. The connection between the nodes represents correlation degree, the thicker the connection, the more frequently the 2 keywords appear together in the same publication. In addition, the color of the nodes represents different clusters namely research topics. As shown in Figure 10, the nodes were assigned different colors according to the needs of the research. It can be seen that the average years of keywords were color-mapped to analyze the evolution of research trends in the field.

It is obviously that the keywords relationship map was divided into 5 clusters. The red and yellow clusters were mainly related to drugs and local anesthesia; while the blue, green and purple clusters were associated with the management of perioperative and postoperative pain. It can be seen that the plane block of the erector spine and the quadratus psoas (QLB) are the hotspots in the research field in recent years.

In addition, for the sake of understanding the specific conditions of keywords, we listed the top 10 high frequency keywords. As shown in Table 7, keywords of ropirvacaine and postoperative anesthesia appeared most frequently. This result indicated that the application of ropivacaine to postoperative anesthesia was the research hotspot in treating postoperative pain.

**Table 7:** Top 10 high-frequency keywords of literatures related to the treatment of postoperative pain with ropivacaine.

Rank	Keywords	Frequency
1	Ropivacaine	596
2	Postoperative analgesia	544
3	Bupivacaine	401
4	Pain	281
5	Surgery	261
6	Analgesia	258
7	Anesthesia	246
8	Efficacy	237
9	Postoperative pain	158
10	Double-blind	156

## 4. Discussion

In this study, we took statistics on the articles (from 2003 to 2022) related to the treatment of postoperative pain with ropivacaine and performed the visualized analysis by VOSviewer. We found that the number of articles published annually is on the rise in this field. This reflects the increasing interest of scholars from various countries to this research field. On the one hand, the management of postoperative pain is an urgent clinical problem and it still needs to discover more effective ways to control postoperative pain. On the other hand, nerve block technology plays a crucial role in treating postoperative pain which can not only alleviate postoperative pain but also reduce the use of opioids. Thus, ropivacaine, as one of the most widely used local anesthetics in nerve block has received more attention from scholars.

By means of statistical analysis on the authors and co-cited authors in the field, we figured out the author who did outstanding contributions to the research and came to a conclusion that the American scholar Ilfeld is the most influential author in the field. Then we looked at the countries that the authors belong to, 4 out of the top 5 authors in the number of published articles are from the United States which shows the influence of the United States in this research field. Finally, by means of visualized analysis of the authors, we concluded that the core authors in this field have formed a relatively stable cooperative group and there is a close relationship between high-productivity authors.

Subsequently, in order to understand the scientific research ability of various countries and institutions in this field, we took an analysis on them. We figured out that the study on the treatment of postoperative pain with ropivacaine has been spread across 59 countries and regions. Although countries have cooperated closely in this field, the overall distribution of research is uneven, and there is a big gap among countries in scientific research field. By the statistical analysis, we learned China has the largest number of articles published in this field, but the total number of citations is not ideal, the average number of citations per article come last in the top 10 countries and has a big gap with other countries, furthermore the quality of the articles published and the influence of the scientific research achievements still need to be improved. The United States ranked second in the number of articles published and first in the total number of citations which reflects the vigour and strong scientific research ability of the United States in this field. It is worth mentioning that Denmark and Canada ranked first in the average number of citations per article. They have great advantages in high citations and high quality literatures. They have made great contributions to the development of this research field. In terms of research institutions, universities are the main force in this field. Among the top 10 institutions, 5 institutions come from the United States and 4 out of 5 institutions are in the universities of the United States; 3 institutions come from China and 1 institution come from Canada and Denmark separately, all of them are in the countries’ universities.

We therefore analyzed the 284 journals the literatures belong to and found that scholars mainly contributed to journals related to anesthesia and pain. According to the number of articles published in the journal, 9 out of the top 10 journals were related to anesthesia and pain. In addition, orthopedic and pharmacy related journals are also the main journals submitted by scholars. The result is similar to the analysis of the co-cited journals. The top 3 co-cited journals are all the top publications in the field of anesthesia, besides, journals of orthopedics and medicine are also the main cited journals.

Finally, we statistically analyzed the keywords, research directions and co-cited literatures. Firstly, we found that ropivacaine and postoperative anesthesia are the 2 most frequently appeared keywords. This indicates that the application of ropivacaine to postoperative anesthesia was a research hotspot in the treatment of postoperative pain. Through the clustering analysis of the keywords, we can see that the keywords in the research field are mainly related to local anesthesia, drugs, and the management of perioperative and postoperative pain. In the meanwhile, through the tag view of the keywords, we can see erector spinal muscle planar block and quadratus psoas muscle block are the latest hot keywords, besides, multimodal analgesia, rhabis abdominis planar block, abdominal plane block, dexamethasone and dexmedetomidine are all popular keywords in recent years. The above analysis reflects the current research development trend be partial to nerve block technology and drug adjuvants. Secondly, the articles in anesthesiology account for more than half of the total articles in this field, which indicates anesthesiology is the main research direction in this field. Thirdly, by analyzing the co-cited literature, we found that the most cited literature was written by Knudsen published in 1997. Knudsen gave a comparison to ropivacaine and bupivacaine, and proposed ropivacaine had lower toxicity than bupivacaine and higher safety for central nervous system and cardiovascular system which provided a theoretical basis for the application of ropivacaine. However, in recent years, the high cited literatures are mainly related to the study of nerve block technology and the analgesic effect of local anesthetic after adding different drug adjuvants. These indicate the changes in the research hotspot and the future development direction in this research field.

This study investigated the current status and development trend of ropivacaine in the last 20 years through bibliometric and visualized analysis, but there are still some limitations in our study: firstly, this study used the WOS core set as the sole source of the data, so there are some omissions in the literatures; secondly, only English literatures were selected for this study, the literatures in other languages were not included.

## 5. Conclusions

In a word, applying bibliometric methods to the study of postoperative pain can not only help physicians conduct comprehensive and intuitive literature statistical analysis, but also understand the current research status in this field, such as which hot topics leading scholars mainly focus on, which new perspectives have been proposed by influential countries, institutions and cooperative groups, and which high-frequency keywords exist in this field, etc. More importantly, mastering this method can help physicians quickly and accurately understand the trend and future development directions in the treatment of postoperative pain with ropivacaine and timely solve urgent clinical problems and propose new treatment ideas for patients.

## Acknowledgments

We would like to thank Dr Hu Zhaolan who is from Xiangya Second Hospital of Central South University for her encouragement and guidance on statistics in this work. The content of acknowledgment was permitted by Dr Hu Zhaolan.

## Author contributions

**Conceptualization:** Bin Yin, Hexiang Wang, Yuanyuan Yu.

**Data curation:** Bin Yin, Liwen Zhang.

**Formal analysis:** Bin Yin.

**Methodology:** Bin Yin, Hexiang Wang.

**Project administration:** Xiaoming Zhao, Yuanyuan Yu.

**Software:** Bin Yin.

**Supervision:** Hexiang Wang, Yuanyuan Yu.

**Visualization:** Liwen Zhang.

**Writing – original draft:** Hexiang Wang.

**Writing – review & editing:** Hexiang Wang, Yuanyuan Yu.
